# Near infrared spectroscopy (NIRS) of the thenar eminence in anesthesia and intensive care

**DOI:** 10.1186/2110-5820-2-11

**Published:** 2012-05-08

**Authors:** Miklos Lipcsey, Nicholas CZ Woinarski, Rinaldo Bellomo

**Affiliations:** 1 Department of Surgery, Section of Anaesthesiology and Intensive Care, Uppsala University, Uppsala, Sweden; 2 Department of Intensive Care, Austin Hospital, Melbourne, VIC, Australia; 3 Australian and New Zealand Intensive Care Research Centre, School of Public Health and Preventive Medicine, Monash University, Alfred Centre, Commercial Rd, Prahran, Melbourne, VIC, 3181, Australia

## Abstract

Near infrared spectroscopy of the thenar eminence (NIRS_th_) is a noninvasive bedside method for assessing tissue oxygenation. The NIRS probe emits light with several wavelengths in the 700- to 850-nm interval and measures the reflected light mainly from a predefined depth. Complex physical models then allow the measurement of the relative concentrations of oxy and deoxyhemoglobin, and thus tissue saturation (StO_2_), as well as an approximation of the tissue hemoglobin, given as tissue hemoglobin index.

Here we review of current knowledge of the application of NIRS_th_ in anesthesia and intensive care.

We performed an analytical and descriptive review of the literature using the terms “near-infrared spectroscopy” combined with “anesthesia,” “anesthesiology,” “intensive care,” “critical care,” “sepsis,” “bleeding,” “hemorrhage,” “surgery,” and “trauma” with particular focus on all NIRS studies involving measurement at the thenar eminence.

We found that NIRS_th_ has been applied as clinical research tool to perform both static and dynamic assessment of StO_2_. Specifically, a vascular occlusion test (VOT) with a pressure cuff can be used to provide a dynamic assessment of the tissue oxygenation response to ischemia. StO_2_ changes during such induced ischemia-reperfusion yield information on oxygen consumption and microvasculatory reactivity. Some evidence suggests that StO_2_ during VOT can detect fluid responsiveness during surgery. In hypovolemic shock, StO_2_ can help to predict outcome, but not in septic shock. In contrast, NIRS parameters during VOT increase the diagnostic and prognostic accuracy in both hypovolemic and septic shock. Minimal data are available on static or dynamic StO_2_ used to guide therapy.

Although the available data are promising, further studies are necessary before NIRS_th_ can become part of routine clinical practice.

## Introduction

Oxygen delivery (DO_2_) and consumption (VO_2_) often are disturbed in critically ill patients [1]. Such disturbance may lead to pathological changes in tissue oxygenation. Thus, monitoring of *tissue oxygenation* appears desirable. Invasive monitoring of systemic DO_2_ and VO_2_ has been used in intensive care medicine for decades [2]. In contrast, no method for assessing tissue oxygenation has yet gained widespread clinical use. This is unfortunate, because tissue oxygenation may reflect changes in the microcirculation, a similarly important target for therapy during major surgery and in the critically ill. Disturbances in microcirculation are common and well documented in hemorrhage and critical illness [3,4], and they logically relate to tissue oxygenation. Thus, monitoring of tissue oxygenation may provide useful information not only about the state of tissue oxygenation itself but probably about the state of the microcirculation. A potentially useful method to monitor tissue oxygenation may be offered by near infrared spectroscopy (NIRS)-based technology.

**Figure 1:**
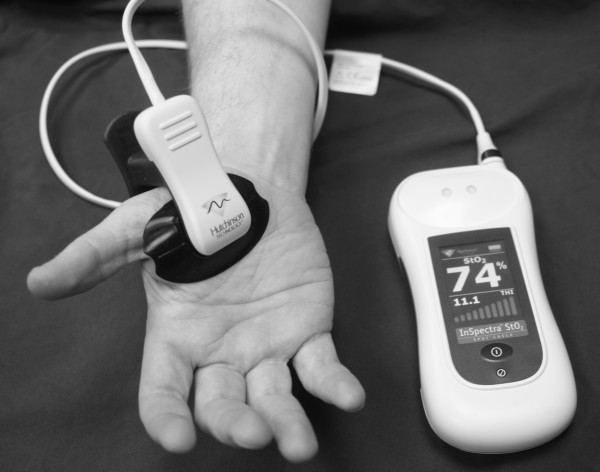
**Portable near infrared spectroscopy (NIRS) monitor in use.** The probe is placed over the thenar eminence.

Although the concept of NIRS has already been available during the second half of the 20^th^ century, its main initial application was for chemical analysis [5,6]. Since the end of the 1970s [7], numerous studies have been published about this method [8,9]. NIRS offers real-time noninvasive monitoring of oxy and deoxyhemoglobin in tissues within a few centimeters from the skin (Figure 1). Furthermore, so-called dynamic values, i.e., values registered during short occlusion of the vascular supply of the area under assessment can be measured. These dynamic values might give additional data on local VO_2_ and probably the condition of the blood flow of the microcirculation [10]. Some NIRS variables also correlate with invasively monitored central circulatory variables [11]. More recently, due to device development, availability, and marketing, clinical research dealing with the utility of NIRS has focused on the specific methodology of NIRS of the thenar eminence (NIRS_th_).

Clinical application of NIRS_th_ remains a relatively new method in a field where no “gold standards” exist. Thus, clinicians considering the use of NIRS_th_ need to understand the principles and evidence behind it and appreciate the many areas of uncertainty that surround its application. In this review, we assess several of these aspects and suggest studies to address areas of controversy.

The principle of clinical NIRS is to noninvasively measure the attenuation of light by hemoglobin, where the emitted light is in a wavelength range longer than visible light [12,13]. NIRS utilizes a narrower spectrum of wavelengths than pulse oximetry, which penetrate deeper into the tissue [14]. Furthermore, whilst NIRS characterizes the total tissue oxy and deoxyhemoglobin in a quantitative and qualitative fashion [13], generating information on oxygen supply and demand, pulse oximetry monitors only hemoglobin in the arterial (pulsatile) part of the local circulation [15].

The near infrared (NIR) light spectrum ranges from 700 nm to 1000 nm. For clinical applications of NIRS, wavelengths approximately 700 to 850 nm are used. The interval stretches on either side of the isobestic point of hemoglobin, i.e., a given wavelength where absorption of light for oxy and deoxyhemoglobin is identical. This wavelength interval maximizes the difference between oxy and deoxyhemoglobin and minimizes the influence of other chromophores, such as myoglobin, cytochrome oxidase, melanin, and bilirubin, on the measurements [16]. Fortunately, the impact of myoglobin on NIRS for measuring tissue oxygenation is minimal [17,18] and melanin, due to its superficial localization, is not a major issue in this regard. Increased conjugated bilirubin levels do influence measurements by dampening the signal. However, trends can still be followed even with jaundice [19].

### **Physical background**

NIRS technology is based on sophisticated physical models, which are greatly simplified in the description below. The attenuation of light in a sample or tissue is proportional to the pathlength of the light and the absorption coefficient of the chromophore according to the physical principles referred to in the Lambert-Beer law [20]. The absorption coefficient of a compound, in the former equation, is a product of the concentration of the compound and the specific extinction coefficient of the compound. Thus, if attenuation of NIR spectrum light is measured and if all other components of the equation described above are known, the concentration of the chromophore, e.g., oxyhemoglobin can be measured. Unfortunately, because the pathlength of light varies due to reflection and interference in a complex milieu of different tissues, absolute concentrations are difficult to estimate. However, the pathlength of light is more or less constant and the extinction coefficients of the common chromophores are known physical quantities. Thus, changes in attenuation of light will be directly proportional to relative changes in concentration of the chromophore. Absolute changes in concentration can be approximated by creating mathematical algorithms for light pathlength in a tissue.

Because the absorption coefficient is direct proportional to concentrations of a chromophore in a tissue studied and extinction coefficient of the compound is constant, estimating the absorption coefficient yields approximations of the absolute chromophore concentration. This is possible through advanced modeling of the behavior of NIR light in tissues and the technical possibility of measuring at several NIR wavelengths [21]. Yet, given the large number of assumptions and approximations in the theoretical basis of NIRS, one may consider trends in different NIRS parameters as more robust than discrete values.

### **Technical considerations**

The NIRS probes in current use measure reflected light. Thus, the NIR light source is placed beside the light sensor. The distance between the light source and sensor determine the distance from where the main part of the reflected light is measured. The technical limit of the monitored depth is the energy of light that does not damage tissues [21]. The main determinants of signal are small vessels of the microcirculation [22].

The brain [23], kidney [24], lower extremity [25], brachioradialis muscle [26], and thenar eminence [27] are all possible sites for bedside NIRS monitoring. The advantage of the thenar eminence compared with other sites, in terms of minimizing variability, is the relatively thin fat tissue over the muscle. Additionally, fibrous strands in its subcutaneous tissue limit the extent of edema formation providing the best possible setting for muscle tissue saturation (StO_2_) measurement even in obese and critically ill patients [28]. Due to anatomical conditions, both the brachioradial muscle and the muscles of the thenar eminence can be easily subjected to the vascular obstruction test (see below).

### **Derived parameters – the vascular occlusion test**

Assessing the ratio of oxy and deoxyhemoglobin in the monitored tissue gives continuous StO_2_. Because absolute hemoglobin content also can be estimated, total tissue hemoglobin and its absolute changes are expressed as tissue hemoglobin index (THI), which can be obtained with this method. THI is, however, not total tissue hemoglobin but its approximation, based on the signal strength of hemoglobin in the monitored area. Low StO_2_ and THI are common findings in hypovolemic shock states.

By occluding the arterial [16] or the venous [29] blood flow to the thenar eminence, NIRS can assess dynamic changes that reflect VO_2_ and postischemic reperfusion and hyperemia. This, vascular occlusion test (VOT), is of special interest in septic shock [30,31] or during anesthesia [32] where the static variables may not be affected despite disturbed circulation.

**Figure 2:**
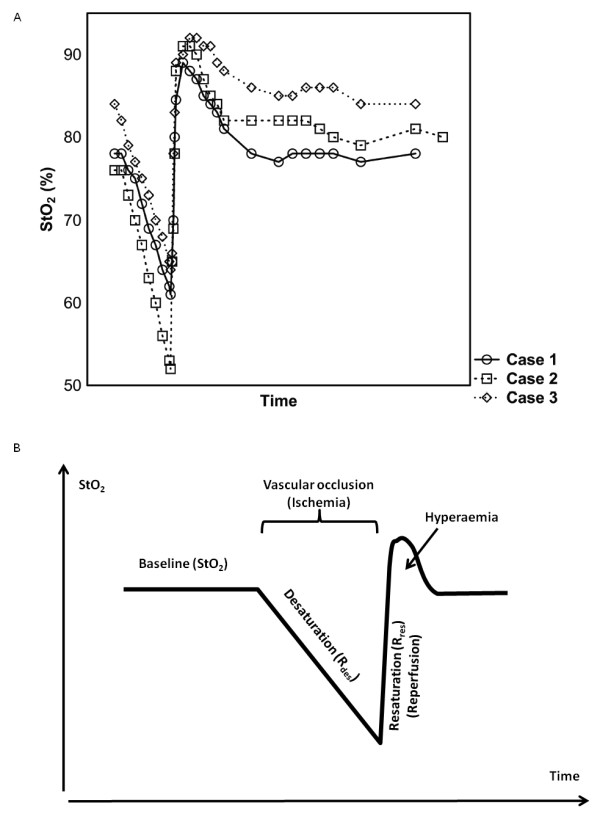
**(a) Typical StO**_**2**_** changes during arterial vascular occlusion test (VOT) in three cases. (b)** Schematic illustration of the StO_2_ changes during VOT.

Arterial and venous vascular occlusion is achieved by a pneumatic cuff on the arm inflated to pressures well above the systolic arterial pressure, aiming to induce ischemia in the thenar muscles and changes in StO_2_ (Figures 2a,b). Considerable duration of obstruction is required to obtain a reperfusion response that differentiates healthy volunteers from resuscitated septic shock patients [33]. However, the optimal way of performing VOT is a matter of debate. Both the intensity and/or duration of the VOT are a matter of controversy; some authors advocate a time-targeted VOT [34,35], and others advocate occlusion to a StO_2_-targeted VOT [33,36]. The argument for time-targeted VOT is that maximal ischemic vascular response is reached within a few minutes of VOT [37] and that long vascular occlusion times could lead to inability to complete VOT procedure due to subject discomfort [38]. On the other hand, argument for StO_2_-targeted VOT, i.e., aiming for StO_2_ of 40%, is that a standardized level of ischemia is achieved, thus interindividual variations in response to VOT giving varying level of ischemia can be minimized.

The rate of desaturation in the thenar muscles (R_des_; % × sec^-1^) after vascular obstruction can be used to estimate VO_2_ in the thenar muscles. The product of the absolute value of R_des_ and mean THI value quantifies the amount of desaturated hemoglobin [16]. The latter can be converted to thenar VO_2_ using the hemoglobin-oxygen binding constant [39].

In addition, after cuff deflation there is a swift restoration of blood flow that can be described in terms of the derivate of the StO_2_ upslope (R_res_; % × sec^-1^). During this reactive hyperemia, the StO_2_ increases over baseline levels, indicating postischemic vasodilatation and capillary recruitment. The integral of the post reoxygenation StO_2_ curve over baseline quantifies reactive hyperemia.

The VOT derived variables add to the robustness of NIRS measurements. In a small study, R_des_, R_res_, and reactive hyperemia were all lower in septic shock patients than in healthy controls [31]. Moreover, R_res_ had an inverse relationship to sequential organ failure assessment (SOFA) and predicted mortality. Finally, a coefficient of variation of less than 10% has been reported for R_res_[33].

Venous occlusion is performed by inflating a pneumatic cuff above venous pressure on the arm [29]. In this setting, NIRS shows increased THI due to vascular congestion and eventually decreasing StO_2_. The venous occlusion method also can be used to estimate local VO_2_. Some have reported varying reproducibility of VO_2_ measurements with venous occlusion [40].

### **NIRS during the perioperative period**

Although there is an extensive literature on NIRS used before [41], during [42-45] or after surgery [46,47], relatively little has been published on NIRS_th_ during the perioperative period. However, in a recent publication R_res_, measured in patients undergoing major abdominal surgery patients, was *decreased* in fluid responsive patients [32]. In the same study, fluid responsiveness was detected by invasive methods, such as pulse pressure variation.

Data are conflicting on the ability of NIRS_th_ to detect blood loss, an area of central interest in anesthesia. In awake volunteers, a 500-ml blood loss at blood donation did not lead to changes in NIRS variables [27]. On the other hand, hemodynamically significant hypovolemia, in awake volunteers, did decrease StO_2_ and THI [48]. A possible explanation could be that tissue hemoglobin and oxygenation at the thenar eminence are not affected by blood loss within the capacity of the compensatory mechanisms of hypovolemia.

In patients after cardiac surgery, StO_2_ and THI did not correlate with global circulatory parameters, but changes in body-finger temperature correlated with changes in StO_2_[49]. However, StO_2_ during the perioperative period in cardiac surgery is lower in patients who develop certain postoperative complications [50] than those who do not [51]. StO_2_ at the thenar eminence does not predict mortality in cardiac surgery [51] or surgical-site infections in colon surgery [52].

### **Intensive care applications**

In a general intensive care population with most patients in resuscitated shock, StO_2_ and R_res_ appear related to capillary refill time and central to peripheral temperature gradient, but not to the etiology of shock [53]. In a mixed group of patients with increased blood lactate levels observed during 8 hours of resuscitation [54], half had low StO_2_ (<70%) on admission. There was no difference in systemic circulatory variables between patients with low or normal StO_2_, but SOFA scores and acute physiology and chronic health evaluation II scores were higher in patients with decreased StO_2_. However, blood transfusion substantially increasing blood hemoglobin did not increase StO_2_ in a mixed group of stable patients [55] and the correlation between blood hemoglobin and tissue hemoglobin index may be limited [31,55]. In patients with hypovolemic or septic patients, more homogenous observations have been described.

### **NIRS in hypovolemic shock**

The rationale for monitoring peripheral tissue as the thenar eminence in hypovolemic shock is centralization of circulation to vital organs leading to decreased blood flow in muscles [56]. In acute hemorrhage, activation of the sympathetic nervous system [57] should decrease thenar muscles blood flow, with increased oxygen extraction and decreased tissue hemoglobin content. In this setting, NIRS_th_ may thus act as a sensor of the vascular response to hypovolemia. In trauma patients with severe shock, StO_2_ is lower in than in milder grades of shock or in normal individuals [58], although patients with shock can present with StO_2_ values as in controls [38].

In a study of severe postpartum hemorrhage, the StO_2_ range overlapped during and after hemorrhage, but StO_2_ increased after control of bleeding [59]. The lowest StO_2_ in the trauma bay has been shown to be as good as the lowest systolic blood pressure at identifying severe shock as defined by experienced clinicians [58]. Furthermore, StO_2_ within 1 hour of admission is lower in trauma patients who develop multiorgan dysfunction (MODS) or die, and a strongest predictor of MODS or death than other diagnostic modalities [60,61]. Low StO_2_ within 1 hour of admission was as sensitive as a high base deficit in identifying patients who developed MODS or died, although specificity for both was low [62]. Finally, low StO_2_ within 1 hour of admission identifies trauma patients who will require blood transfusion within the next 24 hours [63].

The discriminatory power of *dynamic* NIRS parameters, however, is of greater interest. R_res_ was lower in trauma patients compared with controls with little overlap [38]. Moreover, low R_res_ predicted increased troponin I levels in postpartum bleeding [59]. Thus, in hypovolemia, low static StO_2_ predict adverse outcome but dynamic NIRS parameters seem to be more promising.

### **NIRS in sepsis**

In septic shock, although hypovolemia can be a finding [64], there is a substantial microcirculatory disturbance with closed capillaries, arteriovenous shunting, and decreased flow [65]. As a result of these phenomena, oxygen content in the vessels of the microcirculation could be normal, making clinical interpretation of NIRS data complex. These pathophysiologic changes may be not necessarily mirrored by low StO_2_, but rather by low R_res_ and impaired postischemic hyperemic response.

Several studies report a difference in StO_2_ between healthy subjects and patients with severe sepsis or septic shock [16,30,34,66,67], whereas others do not [31,68,69]. Although variation in population characteristics could partly explain these results, in all studies StO_2_ values overlap between septic shock patients and healthy volunteers. This is not surprising because, in sepsis, StO_2_ in sepsis can be at the higher end of the normal spectrum or markedly low [65]. Dynamic NIRS parameters, however, improve the power of the method to distinguish pathologic tissue oxygenation from normal. The R_des_ of the thenar muscles is slower in septic shock patients, indicating a lower rate of tissue VO_2_[16,31,68,69]. Furthermore, R_des_ varies with the severity of systemic infections [69]. Similarly, R_res_ appears lower in septic patients compared with healthy controls [16,30,31,34,70] and decreases with increasing disease severity [30,31]. R_res_ ranges overlap minimally [30,68,70] or not at all [34] when comparing healthy controls and septic patients, and R_res_ improves as septic shock resolves [34]. Finally postischemic hyperemia is decreased in septic patients compared with healthy controls [30,31]. Thus, NIRS_th_ could be a method for bedside assessment of the microcirculation [71].

Monitoring global hemodynamics with NIRS_th_ also has attracted interest. In sepsis, treatment based on venous saturation in the superior vena cava (ScvO_2_) [64] or the pulmonary artery (SvO_2_) [72] is used, because these are markers of global DO_2_ and VO_2_ balance [73]. Noninvasively obtained surrogates of ScvO_2_ or SvO_2_ would be valuable. In this regard, StO_2_ correlates to ScvO_2_[74] and SvO_2_[67] in patients with severe sepsis or septic shock; however, correlation coefficients are relatively low. The accuracy of estimating SvO_2_ could be substantially improved by calculating the “NIRS-derived SvO_2_” [67]. In severe sepsis and severe heart failure, StO_2_, however, did not estimate SvO_2_[75]. Still, data suggest that patients with severe sepsis or septic shock and low StO_2_ also have low ScvO_2_, suggesting hypodynamic circulation [74].

Variables related to DO_2_ may correlate with NIRS parameters. R_res_ correlates with cardiac output and to a lesser extent with blood lactate levels in septic patients [34], whereas StO_2_ does not correlate with lactate or base deficit [67]. A low StO_2_ predicts a very low DO_2_ in early sepsis with high sensitivity and specificity [76]; however, moderately low DO_2_ does not correlate with StO_2_. Neither was change in R_res_ correlated to change in cardiac output in septic patients. Finally, StO_2_ did not correlate with the severity of illness [67], but a StO_2_ <78% in resuscitated patients predicted mortality [77]. Low R_res_ correlates with organ failure [31] and R_res_ is lower in nonsurvivors than survivors [34].

Recently, it has been reported that increasing blood pressure with noradrenalin infusion from 65 mmHg to 85 mmHg in resuscitated sepsis patients normalized R_res_[78]. These patients, although seemingly resuscitated according to the Surviving Sepsis Campaign guidelines, could improve the thenar perfusion by achieving higher mean arterial pressure [79]. These data could suggest that NIRS can identify patients who benefit from treatment beyond the traditional goals, thus the usefulness of NIRS as a bedside tool to optimize tissue oxygenation [71]. Thus, in resuscitated, septic patients, dynamic NIRS of the thenar eminence provides information on microcirculation and trends could be used to guide treatment.

### **NIRS in miscellaneous conditions**

In patients with chronic heart failure, thenar StO_2_, R_des_, and R_res_ are low [80]. NIRS_th_ parameters in these patients improved after 6 hours of dobutamine or levosimendan infusion [80], or 3 months of regular exercise training [81]. Patients with cirrhosis demonstrated a supranormal hyperemic response after vascular occlusion test [82], which increases with increasing severity of liver disease [82].

### **NIRS-derived and central hemodynamic parameters**

The performance of NIRS_th_ in estimating global circulatory parameters is highly dependent on the coupling between the circulation of the hand and the central circulation. Static NIRS parameters, such as StO_2_, should, in theory, be related to centrally measured circulatory parameters. However, this relationship has not been demonstrated in general in critically ill patients [54] or after cardiac surgery [49]. Although, global venous saturations have been described to correlate to StO_2_ in sepsis, this relationship is weak [67,74,83]. In sepsis, correlation between StO_2_ and SvO_2_ can be improved with correction equations [67]. Only substantial deviations from normal DO_2_ levels are detected reliably by StO_2_ in sepsis [76]. In these patients, R_res_ correlates with cardiac index and blood lactate levels [34].

Low StO_2_ also predicts MODS in a mixed population of critically ill patients [54], in trauma patients [60-62], and in postcardiac surgery patients [51]. In sepsis, dynamic NIRS variables, such as R_des_[69] and R_res_[31,34], have been associated with organ failure.

### **Limitations**

NIRS_th_ monitors peripheral muscle as a marker of perfusion elsewhere. The theoretical concern is whether the small volume of distal muscle can be a good indicator of the state of the tissue oxygenation in the rest of the body and the vital organs. For example, local factors, such as obstruction to flow by atherosclerosis, an arterial catheter, or thrombosis after previous arterial catheterization, could affect measurement. Although a brief report suggests that catheterization of the radial artery in adult, elective, surgical patients does not affect StO_2_[84], this may not be the case in critically ill patients with circulatory failure.

StO_2_ is not the same as tissue blood flow in the tissue or even oxygen supply. StO_2_ is affected by local VO_2_, which could be affected by states that alter muscle metabolism, such as muscle relaxants. The balance between the metabolic state of the muscle and other vital organs may vary between individuals and intraindividually during the course of disease, affecting the global relevance of some of the NIRS variables. Also tissues overlaying the thenar muscles can influence measurements [85]. Very dark skin with high melanin content or thick, edematous, or injured connective tissues and low hemoglobin levels also could pose problems with NIRS measurements [86].

There are many studies on NIRS in the perioperative period and critical illness where many different sites are monitored. Moreover, the methodology of NIRS is not standardized [87,88]. Several probes are available on the market, different occlusion protocols are used, and different parts of downslope and the upslope StO_2_ curves at vascular occlusion are used in calculations. Furthermore, studies are conducted on patients in different phases of disease, which may represent different pathophysiologic situations. Hence, comparing results can be difficult (Table 1).

**Table 1:** **Summary of tissue saturation (StO**_**2**_**), desaturation rate (R**_**des**_**), and resaturation (R**_**res**_**) values in different shock states in the literature**

	**Study**	**Controls**	**Severe sepsis/septic shock**	**Hemorrhagic shock**
**StO**_**2**_** (%)**	Skarda et al. [16]	75 ± 15	87 ± 6	
	Creteur et al. [30]	80 ± 7	72 ± 11	
	Doerschug et al. [31]	84 ± 10	82 ± 13	
	Mayeur et al. [33]	82 ± 4	80 ± 10	
	Nanas et al. [68]	82 ± 6	76 ± 17	
	Pareznik et al. [69]	83 (79–93)	89 (65–92)	
	Georger et al. [70]	82 ± 4	75 ± 9	
	Podbregar et al. [75]	84 ± 4	90 ± 7	
	Crookes et al. [58]	87 ± 6.4		45 ± 27
	Gómez et al. [38]	88 ± 5		86 ± 9
**R**_**des**_** (%/s)**	Mayeur et al. [33]	0.18 ± 0.05	0.16 ± 0.06	
	Nanas et al. [68]	0.6 ± 0.18	0.25 ± 0.1	
	Pareznik et al. [69]	0.62 (0.46-0.94)	0.12 (0.06-0.18)	
	Georger et al. [70]	0.22 ± 0.05	0.16 ± 0.07	
	Gómez et al. [38]		0.17 ± 0.06	0.15 ± 0.09
**R**_**res**_** (%/s)**	Skarda et al. [16]	3.3 ± 0.7	2.3 ± 1	
	Creteur et al. [30]	4.8 ± 1.6	2.3 ± 1.3	
	Doerschug et al. [31]	4.7 ± 1.1	2.3 ± 1.5	
	Mayeur et al. [33]	5.4 ± 1.1	2.3 ± 1.4	
	Nanas et al. [68]	12 ± 3.7	2.4 ± 1.7	
	Georger et al. [70]	2.3 ± 0.5	1 ± 0.6	
	Gómez et al. [38]	5.4 ± 1.3		2.5 ± 1.3

Data are given as mean ± SD or median (range).

Static NIRS variables are influenced by the temperature of the hands compared with core temperature [53] and could be a reason for the overlap between patients with normal and abnormal peripheral circulation. In anesthesia with volatile anaesthetics, vasodilatation is a common feature [89] and monitoring StO_2_ would be expected to be less affected by peripheral vascular tone.

The question of whether organ failure in sepsis is mostly dependent on disturbed mitochondrial oxygen metabolism [90-92], or on limited DO_2_[93,94], is a matter of debate. NIRS does not assess mitochondrial oxygen metabolism in sepsis, although decreased tissue VO_2_, R_des_, could imply mitochondrial dysfunction. However, decreased tissue VO_2_ also could depend on shunting of delivered oxygen [65]. This phenomenon is not measured by NIRS either. However, with all of its limitations, NIRS_th_ is a noninvasive method that, given that supporting data will be available, could become part of anesthetic and intensive care monitoring in a manner similar to pulse oximetry.

Conclusions NIRS_th_ eminence estimates StO_2_ in peripheral muscles. In hypovolemia, such StO_2_ is decreased and relates to severity of disease and outcome. Hence, StO_2_ measurements could aid with the clinical management of these patients. In unresuscitated or inadequately resuscitated septic shock, R_des_ and R_res_ are decreased, indicating disturbed tissue oxygen metabolism and microvascular reserve. The pathologic findings in these dynamic NIRS parameters could be of value in resuscitated sepsis where macrocirculatory failure has been corrected and during anesthesia to monitor adequacy of peripheral perfusion and fluid status. Despite its limitations, NIRS_th_ takes monitoring from global to local level. The existing literature on NIRS_th_ is mainly focused on validation of this technique. Future studies that implement NIRS_th_ into treatment algorithms in anesthesia and intensive care would be valuable to define the place for this monitoring modality in daily management of critically ill patients.

## **Competing interests**

The authors declare that they have no competing interests.

## **Authors contributions**

All authors conceived the manuscript. Both ML and NCZW reviewed the literature. ML wrote the first draft. RB reviewed and refined the first draft. Further drafts were completed by ML, RB and NCZW. All authors read and approved the final manuscript.
